# Management of Acute Ferrous Sulfate Poisoning Using Activated Charcoal Monotherapy: A Case Report

**DOI:** 10.7759/cureus.49972

**Published:** 2023-12-05

**Authors:** Salah Eldin A Abdel Haleem

**Affiliations:** 1 Department of Pharmacology and Therapeutics, Faculty of Medicine, University of Bahri, Khartoum, SDN; 2 Department of Pharmacology, Faculty of Medicine, Al-Baha University, Al Baha, SAU

**Keywords:** adolescent, attempted suicide, activated charcoal, toxicodromes, ferrous sulphate

## Abstract

A 13-year-old schoolgirl presented to a public hospital having ingested over 70 tablets of 65 mg of prescription ferrous sulphate within the last hour. She complained of a 7/10 sharp abdominal pain. Signs included lethargy, agony, diaphoresis, and frequent eructation. Gastrointestinal movements were audible and frequent. Heart rate was at 50 beats per minute with intact but weak pulsations. Blood pressure was at 95/65 mmHg. During the clerking, she started vomiting in pouts separated by few-minute intervals. The vomitus was watery, yellowish, and had a rusty-ironic smell. Serum iron was at 200 mcg/dL and 230 mcg/dL at the time of admission to the hospital and the time of discharge, respectively. The patient received intravenous Ringer’s lactate solution. She was detoxified by the chewing of a single dose of 100 g activated charcoal. She stayed in remission throughout the follow-up. It was concluded that monotherapy with oral activated charcoal arrests the progress of the toxicodrome.

## Introduction

The most popular iron formulation is the 325 mg ferrous sulfate tablet that contains 20% (or 65 mg) of elemental iron per tablet [1]. Ingestion of more than 60 mg/kg of elemental iron can result in severe toxicity and lead to severe morbidity and mortality [1,2]. In 2015, in the United States, 1161 cases of acute iron poisoning were treated in a healthcare facility and there was one death [3]. Serious iron poisoning is particularly seen in young children after ingestion of medicinal adult iron preparations. Despite the ill definition of a standard toxic dose, significant gastrointestinal manifestations occur following the ingestion of 20 mg/kg of body-weight of elemental iron [2].

The pathophysiology of acute iron toxicity is corrosive or cellular. It can cause caustic injury to the gastrointestinal mucosa clinically presenting as nausea, vomiting, abdominal pain, and diarrhea. Significant fluid and blood loss can present with symptoms of hypovolemia. Hemorrhagic necrosis of gastrointestinal mucosa and/or perforation can clinically present with hematemesis and symptoms of peritonitis. Free iron impairs cellular metabolism in the heart, liver, and central nervous system. It concentrates in the mitochondria disrupting oxidative phosphorylation, catalyzing lipid peroxidation, forming free radicals, and eventually leading to apoptosis [4]. Widespread cellular injury presents clinically as metabolic acidosis [1].

The toxicokinetics involves peaking of serum iron level at two to four hours post ingestion, 10% of which binds transferrin. The normal range of serum iron is 50-150 micrograms/dL, and total iron-binding capacity (TIBC) ranges from 300-400 micrograms/dL. When transferrin becomes saturated, excess free iron circulating in the plasma is directly toxic to target organs [5]. Acute overdose iron toxicity typically runs a five-stage clinical course [1]. Stage 1 (0.5-6 hours) clinically presents with gastrointestinal symptoms of abdominal pain, vomiting, diarrhea, hematemesis, and hematochezia. In stage 2 (6-24 hours), the patient shows signs of temporary remission. Stage 3 (6-72 hours) witnesses the recurrence of gastrointestinal symptoms, shock, metabolic acidosis, iron-induced coagulopathy, hepatic dysfunction, cardiomyopathy, and renal failure. In stage 4 (12-96 hours), signs of liver failure are exhibited by liver enzyme elevation. Pyloric or proximal bowel scarring and obstruction secondary to healing of the injured gastrointestinal mucosa characterize stage 5 (two to eight weeks) [1]. Be that as it may, not every patient runs the same course of the toxicodrome [1]. A patient may present in, or skip, any of the five stages.

Staging should be based on symptoms and clinical manifestations and not on the time of ingestion [4]. The most useful laboratory serum iron level investigation is recommended at four to six hours after ingestion, followed by a six to eight-hour post-ingestion measure. Levels in excess of 500 micrograms/dL warrant severe systemic toxicity. Deceptively low iron levels after its peak are due to rapid iron clearance from the serum to deposition in the liver [1]. Although laboratory investigations routinely include electrolytes, kidney function, serum glucose, coagulation studies, complete blood count, and liver functions, plain radiographs may reveal iron in the gastrointestinal tract if the ingested iron preparations are radiopaque. Despite the pathognomonic nature of such a finding, there is no correlation between radiopacities on X-rays and the severity of poisoning [6].

The mainstay of management is stabilizing vital functions, and removing unabsorbed iron from the gastrointestinal tract. Intravenous deferoxamine is indicated when there are serious clinical symptoms or when a serum iron level exceeds 500 micrograms/dL within eight hours post ingestion. Timely therapeutic intervention results in a favorable prognosis [2]. Despite the previous common belief that activated charcoal does not appreciably adsorb iron salts, earlier in vitro work indicates some adsorption of iron [7]; activated charcoal adsorb ferrous sulfate to a greater extent at pH environments where iron is typically absorbed from the gastrointestinal tract. These results indicate that activated charcoal may prove an effective therapy for acute iron poisoning [7]. In the present report, a 13-year-old schoolgirl presented to the ER with the suspicion of ingesting over 70 tablets of prescription ferrous sulfate in the past hour and with symptoms highly suggestive of acute overdose iron toxicity.

## Case presentation

A 13-year-old schoolgirl was presented by her mother to the ER. The mother suspected that her daughter had ingested over 70 tablets of 65 mg of ferrous sulfate. She presented the empty 100-tablet pharmaceutical dosage form container which she received as a prescription less than a month ago and from which she had been taking one tablet on a daily basis. The presenting complaint was acute abdominal pain for the past hour. She found her daughter an hour after return from school in discomfort, reluctant to leave her bed or to communicate in articulated speech as usual, nauseated with frequent retching and salivation, and sweating all over with beads over her forehead. Thirty minutes after this, the daughter developed abdominal pain. The mother found the empty ferrous sulfate container on the table beside the bed.

Case history

The patient described her pain as a sharp pain all over the abdomen, which was not radiated to the shoulder, jaw, or any other part of the body, and that its onset had gradually escalated within the past 30 minutes. On a scale from 1-10, she described the pain as grade 7. Due to the emergency of her case, she did not attempt a relieving analgesic treatment. Sitting and standing postures were reported as exacerbating compared to the recumbent posture. The patient was not suffering from any somatic or mental illness and nor was she in any apparent depressive status. There was no history of self-induced, drug-induced, or psychogenic vomiting. History was negative regarding eating disorders or major psychiatric diseases. Her past medical history was insignificant with no history of chronic illness, admission to hospital, blood transfusion, or surgical operation. There was no significant family history of a chronic illness or mental illness. None of the family members or their relatives had mental illness or committed suicide. The patient was not on any current or continuous medication. Regarding social history, both parents were middle socioeconomic-class elementary school teachers with two daughters, a 15-year-old secondary school student and the patient, who was in the final class of elementary school. Both daughters reached puberty at 13 years of age. There was no family history of home violence or disagreements, although the two daughters had a few altercations now and then.

Physical examination

By observation, the teenager had a good physique and weighed around 55 kg, with signs of agony and discomfort with apathy, diaphoresis, pallor, salivation, frequent spitting, and eructation. On examination, the patient was conscious with a Glasgow Coma Scale score of 15 but lethargic. Neurologic examination revealed intact cranial nerves and no abnormality in the peripheral nervous system. Cardiopulmonary examination revealed bradycardia with the heart rate at 50 beats per minute with intact but weak pulsations. Blood pressure was at 95/65 mmHg showing hypotension and the respiratory rate showed bradypnea at 14 breaths per minute. The patient was not in shock. Abdominal examination revealed excessive gastrointestinal movements with audible frequent bowel sounds. There was no hepatomegaly or splenomegaly with a normal liver span and no abdominal distention. Muscles of the abdominal wall were tense and were overlaid with diaphoretic skin and mild tenderness. Genito-urinary system examination was insignificant. Prior to any further examination investigations, the patient started spontaneous vomiting. Vomiting was not projectile but came out in pouts separated by few-minute intervals. The vomitus was watery, yellowish, and with a characteristic rusty-ironic smell that could be sensed in the medical ER for the rest of the day despite repeated cleaning of the floor and spraying of a fragrancing aerosol. Be that as it may, there was no hematemesis, the vomitus was not blood-streaked, not coffee ground, not bile-stained, nor contained fecal material. After four and six hours, the patient had two loose motions. Defecation and micturition were not associated with dyschesia or tenesmus. Stools were offensive and associated with passing flatus but without hematochezia; urine was likewise normal. It was noticed that the patient was intense and emotionally vulnerable. She communicated her apprehension and feeling of guilt to her mother, and she explicitly expressed her sorrow on the instance of her vomitus contaminating the medical ER floor in front of her couch.

Biochemical tests and laboratory investigations

The patient’s history and her clinical presentation, particularly the characteristics of emesis, rolled out differential diagnoses other than acute overdose iron toxicity. Investigations were thus limited to routine complete blood count and blood iron levels at zero, four, and eight hours and liver function tests. Urine iron level investigations were not available in the hospital at that time. The results of these investigations are shown in Tables 1-4.

**Table 1:** Complete blood count results at the time of presentation

Test	Result	Reference range
White blood cells	9,000 cells/mcL	4,500-11,000 cells/mcL
Red blood cells	4.56 cells/mcL	4.56-5.96 cells/mcL for men; 4.16-5.16 cells/mcL for women
Hemoglobin	15 gm/dL	14-17.5 gm/dL for men; 12.3-15.3 gm/dL for women
Hematocrit	45%	41.5-50.4% for men; 35.9-44.6% for women
Mean corpuscular volume	88	80-96
Platelets	190,000 platelets/mcL	150,000-450,000 platelets/mcL

**Table 2:** Serum iron levels at the time of admission to hospital

Test	Result	Reference range
Serum Iron concentration	200 mcg/dL	70-175 mcg/dL for men; 50-170 mcg/dL for women; 50-120 mcg/dL for children
Serum transferrin saturation	50%	< 45%
Total iron-binding capacity (TIBC)	270 mcg/dL	250-450 mcg/dL for men and women

**Table 3:** Serum iron levels at the time of discharge from hospital

Test	Result	Reference range
Serum Iron concentration	230 mcg/dL	70-175 mcg/dL for men; 50-170 mcg/dL for women; 50-120 mcg/dL for children
Serum transferrin saturation	50%	< 45%
Total iron-binding capacity (TIBC)	310 mcg/dL	250-450 mcg/dL for men and women

**Table 4:** Liver function tests

Test	Result	Reference range
Alanine transaminase (ALT)	35 U/L	7- 55 units per liter (U/L)
Aspartate transaminase (AST)	25 U/L	8-48 U/L
Alkaline phosphatase (ALP)	60 U/L	40-129 U/L
Albumin	3.6 g/dL	3.5-5.0 grams per deciliter (g/dL)
Total protein	6 g/dL	6.0- 8.3 grams per deciliter (g/dL)
Bilirubin	0.7 mg/dL	0.1- 1.2 milligrams per deciliter (mg/dL)
Gamma-glutamyltransferase (GGT)	24 U/L	8- 61 U/L
L-lactate dehydrogenase (LD)	116 U/L	122- 222 U/L
Prothrombin time (PT)	10 seconds	10- 13 seconds

Management plan

Having reached a definitive diagnosis and excluded other differential diagnoses and/or co-morbidities, a management plan was established based on standard treatment guidelines (STGs). The therapeutic goal was to achieve the patient’s detoxification and prevention of complications. It focused on a stepwise approach to maintain the stability of the patient’s clinical status by preventing dehydration and/or hypotension by administering intravenous Ringer’s lactate solution, to remove the offender drug overdose from the patient’s gastrointestinal tract by assisting the eminent vomiting, preventing aspiration of the vomitus, and administering of a single dose of 100 g activated charcoal, and to monitor the blood iron level and administer intravenous deferoxamine if the serum iron level reaches 500 micrograms/dL, a level that can cause target organ damage. The monitoring plan was set to follow the patient's clinical parameters continuously and follow the serum iron levels at zero hours, four hours, eight hours, 12 hours, and at the time of discharge from the hospital. Liver function tests were scheduled at zero hours if the iron level exceeded 500 micrograms/dL, and prior to discharge from the hospital. Urine iron level investigation and liver biopsy were also planned in case the liver functions deteriorate.

Expected outcome of the treatment plan

In spite of the large dose of iron ingested, a good prognosis was expected considering the early presentation of the case, the young age of the patient, her excellent physical condition, and the absence of co-morbidities. It has been proposed that, within 24 hours of treatment and follow-up, she would be cured without further complications. Be that as it may, adverse sequelae, although not anticipated with robust case attendance and follow-up, could not be completely rolled out at that time.

Actual outcome

Emergency management included assisting the patient to assume a position that assisted her vomiting without aspiration of the vomitus, establishing an intravenous line using a large bore cannula, administration of Ringer’s lactate solution, and chewing 100 g of activated charcoal. This was within 20 minutes after admission. Administration of intravenous deferoxamine was considered in the initial treatment plan according to the progression of the case and was abandoned as the patient remained in remission at the 12-hour evaluation. The patient was referred for psychiatric evaluation. She was discharged after 24 hours stay in the hospital. The follow-up visits, scheduled at 24 hours, 48 hours, and seven days later, showed that the patient remained in remission. Clinical examinations and investigations were all normal. Throughout her stay in the hospital, the patient expressed her sorrow for putting her family and the physicians into this trouble. With time, she was very cooperative and showed that she was willing and able to circumvent this crisis.

## Discussion

Although the relatively high incidence of adult iron pharmaceutical dosage leads to intoxication among children [1] and gives the present case some importance, other identifiers contribute more robustly to the importance of this case. These include the exposure to the largest possible dose that can be obtained from a pharmaceutical dosage form, and the novel achievement of remission and cure on activated charcoal monotherapy. The 100 small tablets of ferrous sulfate containers are the most popular iron pharmaceutical dosage form in Sudan. These are supplied, free of charge, courtesy of the reproductive healthcare program at primary healthcare centers. This gave the patient the opportunity to consume more than 70 tablets of this drug. Mathematically speaking, the patient was challenged by the exposure to an excess of 82 mg per kilogram of her body weight. This exceeds the defined limit of 60 mg/kg of body weight for dangerous prescription iron exposures [1,2]. It by far exceeds the 20 mg per kilogram of body weight dose limit enough to produce signs of toxicity in pediatric patients [2].

The severity of the gastrointestinal symptoms and lethargy in the presenting complaint of the present case is the expression of this high overdose exposure. This is consistent with the typical clinical picture of acute iron toxicity [4,1]. These symptoms would have been expected to exponentially escalate with regard to the reported toxicokinetics and toxicodynamics of the toxicodrome, particularly at such a large dose of the offender drug [4,1,5]. In order of importance, the progress of the toxicodrome through stages 1 to 5 is a function of dose, the patient’s condition, the time of medical attention post exposure, and the quality of the therapeutic interventions [2]. The present case rearranges these attributes to prioritize the patient’s condition and medical intervention over the attribute of dose of exposure.

The robustness of the patient in this case and her will to survive the crisis, contributed to the final favorable therapeutic outcome. The management plan, which followed the standard treatment guidelines, was tailored to a graded response that adhered to the progress of the case through the stages of the toxicodrome. The adequacy of oral activated charcoal in detoxifying the patient is a novel outcome. It is consistent with a previous report, which investigated the in vitro potential of activated charcoal to adsorb and chelate iron [7]. It renders the present case as the novel evidence-based medical report of this monotherapy of acute overdose pharmaceutical iron toxicity in hospital settings. The therapeutic interventions were guided by a series of pathological investigations based on the standard treatment guidelines, and the clinical progress of the case. Abdominal radiographs, consistent with previous evidence-based reports [6], are not diagnostic. Hence, they were not performed.

Finally, the present case conveys an evidence-based message that corroborates with the existing evidence regarding the presentation and the possible progress of acute overdose pharmaceutical iron toxicodrome, but it detracts from current evidence regarding the inadequacy and/or lack of efficacy of oral activated charcoal in detoxifying the patient and achieving cure. This evidence opens a vista of value for future clinical practice.

## Conclusions

Acute overdose pharmaceutical iron toxicity can run any of the five stages of a toxicodrome; and the course of the toxicodrome depends largely on the patient’s condition, in addition to exposure and medical interventions. Monotherapy with oral activated charcoal can adequately detoxify the patient to arrest the progress across the stages of the toxicodrome. Robust medical attention and patient follow-up are needed.
